# Unequal Impact of COVID-19 on Private and Academic Neurosurgical Workforce: Results of an International Survey

**DOI:** 10.3389/fsurg.2021.749399

**Published:** 2021-10-01

**Authors:** Sami Ridwan, Mario Ganau, Cesare Zoia, Marike Broekman, Alexander Grote, Hans Clusmann

**Affiliations:** ^1^Department of Neurosurgery, Klinikum Ibbenbueren, Ibbenbueren, Germany; ^2^Department of Neurosurgery, Oxford University Hospitals NHS Foundation Trust, Oxford, United Kingdom; ^3^Department of Neurosurgery, Fondazione IRCCS Policlinico San Matteo, Pavia, Italy; ^4^Department of Neurosurgery, Haaglanden Medical Center, The Hague, Leiden University Medical Center, Leiden, Netherlands; ^5^Department of Neurology, Massachusetts General Hospital, Boston, MA, United States; ^6^Department of Neurosurgery, Bethel Clinic, Bielefeld, Germany; ^7^Department of Neurosurgery, RWTH Aachen University, Aachen, Germany

**Keywords:** neurosurgery, COVID-19, SARS-CoV-2, pandemic, EANS

## Abstract

**Background:** Since the COVID-19 outbreak several manuscripts regarding neurosurgical practice during this pandemic have been published. Qualitative studies on how the pandemic affected neurosurgeons, with additional focus on their practice, are still scarce. This study's objective was to investigate the impact of COVID-19 on various aspects of the professional and private life of a homogeneous group of international neurosurgeons affiliated to the European Association of Neurosurgical Societies (EANS).

**Methods:** Neurosurgeons from Europe and abroad were invited to participate in an online survey endorsed by the Individual Membership Committee of the EANS. The survey captured a subjective snapshot of the impact of the first wave of the COVID-19 pandemic on EANS members and was advertised through its Institutional website. In addition to departmental data, personal feeling of safety, financial security, local precautions, number of surgeries performed, changes in daily routine, and other practice-related information were inquired. Differences among practice types were closely reviewed.

**Results:** The survey was distributed between April and May 2020: 204 neurosurgeons participated. Participants were typically active EANS members (73%), consultants (57.9%), from university hospitals (64.5%). Elective surgical practice was still ongoing only for 15% of responders, whereas 18.7% of them had already transitioned to COVID-19 and emergency medical services. While 65.7% of participants thought their institutions were adequately prepared, lack of testing for SARS-CoV-2, and scarcity of personal protective equipment were still a matter of concern for most of them. Overall surgical activity dropped by 68% (cranial by 54%, spine by 71%), and even emergencies decreased by 35%. COVID-19 prompted changes in communication in 74% of departments, 44% increased telemedicine by >50%. While most neurosurgeons had concerns about personal and families' health, financial outlook appeared to be gloomy only for private practitioners.

**Conclusion:** The lockdown imposed in many countries by the COVID-19 outbreak called for immediate modification of working routine and resulted in a dramatic decrease of elective surgical procedures. Neurosurgeons share common concerns but were not equally exposed to the personal health and financial dangers of the ongoing pandemic.

## Introduction

The COVID-19 outbreak, and the reactive measures implemented in many countries, imposed a number of challenges to neurosurgeons: from inconsistent flow of information over time, to deviation from well-defined standards of care (1, 2).

Due to the variety of national and international political measures dictated by the nature of the pandemic, and the different timescale of diffusion of SARS-CoV-2 throughout the world, neurosurgeons were flooded with heterogeneous regulations, protocols, and timely produced standard operating procedures (3–17).

These measures obliged to a deviation from well-defined, and largely agreed upon, standards of care. In extreme, yet not uncommon, situations neurosurgeons had to face the challenges of rationing healthcare provision, likely to result in higher mortality or less favorable outcomes, especially in cancer patients (18).

For some neurosurgeons, clinical practice changed substantially when they were redeployed to intensive care units (ICU) to assist critically ill patients requiring mechanical ventilation. Neurosurgeons who got involved into this drastic hospital reorganization ended up fulfilling duties for which most of them had not been formally trained in their career, a situation never seen before during peacetime periods (19).

While all the above represented a source of concerns, particularly about quality of patients' care, neurosurgeons were also affected by personal issues, as much as all other human beings. Those issues included personal security, but also families' and colleagues' health and wellbeing. Last but not least, the concerns regarded the economic and financial consequences of this crisis, particularly for all those neurosurgeons working in private practice.

So far, the specific impact of the COVID-19 pandemic on the life of neurosurgeons has not been analyzed through qualitative research (1). Hence, this study's objective was to investigate the impact of COVID-19 on various aspects of the professional and private life of a homogeneous group of international neurosurgeons affiliated to the European Association of Neurosurgical Societies (EANS). The EANS, which accounts for over 1,900 members (according to the most recent membership's report issued on August 2020), including 181 international colleagues from continents other than Europe, and 276 from low- and low-middle income countries, has looked into the heterogeneity of recommendations, political regulations, and variation in the pandemic's scale among EANS members' countries. The EANS Individual Membership Committee (EANS IMC) designed an online survey to investigate the challenges that neurosurgery as a specialty, and neurosurgeons as healthcare professionals and individuals, have faced during the initial wave of the pandemic. By collecting their witnesses, this study helped understanding the role of EANS towards its international neurosurgical community and anticipating the long-term effects of the pandemic.

## Methods

A questionnaire consisting of forty-two questions was drafted to timely assess the relevant issues outlined above. The research goal was to investigate and record the impact of the COVID-19 outbreak on global neurosurgeons through acquisition of qualitative data. The questionnaire was prepared and distributed using the online platform SurveyMonkey (www.surveymonkey.com, SurveyMonkey Inc., San Mateo, California, USA). The design and internal validation stage was conducted during the second half of March 2020, and the survey was opened to responders on April 3^rd^, 2020. The survey was promoted by the EANS, its official sponsor, and distributed via E-mail to its members; additionally, a weblink to the survey was continuously displayed on the EANS Institutional website for a total of five consecutive weeks. Neurosurgeons receiving the survey were encouraged to inform colleagues and non-members by disseminating the weblink via social media and other means of interpersonal communication. Data evaluation was started on May 18^th^, 2020. The estimated completion rate according to SurveyMonkey was 52%. This study was intended to reach at least comparable response rate (3 to 5% when calculated using standard distribution platforms excluding social media) and completion rate (70%) to recently published neurosurgical surveys (20–22).

### Local and Departmental Data

Responders were asked to provide information regarding their neurosurgical practice by selecting the country and city of work, the number of neurosurgical departments in the city, the size of their own department (including number of beds and average surgical procedures per year), and their personal occupation, such as role and diversified practice (either academic, public or private hospital types). The terms trainee and resident were both used in this survey to overcome possible international deviations regarding training programs. Hospitals were divided in 5 groups to cover national and international conditions: university hospitals, maximum care hospitals also known as non-academic tertiary hospitals, military hospitals, town hospitals also known as general hospitals, and private hospitals and clinics. With respect to the COVID-19 pandemic, responders were asked to provide the average number of neurosurgical interventions performed from the start of the outbreak up until the completion of the survey. As such, the survey collected departmental data with the breakdown of cranial, spine and emergency procedures before and during the pandemic. Also, termination (and/or reduction to a minimum) of elective surgical and outpatient services, rotation of neurosurgical personnel to Emergency Department (ED) or ICU services, and rate of COVID-19 infected personnel were surveyed.

### Individual Perception

Beside departmental data, the questionnaire was intended to capture the neurosurgeons' individual perception of safety, financial concerns, and personal opinions regarding political measures implemented in their countries, as well as organizational changes implemented in their local institutions. Specific questions were designed to capture if and to what extent neurosurgeons were ready and adequately able to take up duties in ED or ICU. Other questions investigated neurosurgeons' perception of their patients' understanding, and their colleagues' attitude towards this novel situation.

The full-version questionnaire including multiple choice and free text options is shown in Supplementary Data (see Supplemental Digital Content).

### Data Analysis

Anonymized data analysis was performed utilizing SPSS 25.0 for Mac (IBM Corp. Released 2017. IBM SPSS Statistics for Mac, Version 25.0. Armonk, NY: IBM Corp.). Prior to data analysis, free text passages were thoroughly reviewed for typing and form errors with possible impact on software analysis and were properly aligned. Numerical scale slider answers (0–100) were used to identify 4 groups of responders depending on the individual perception of the issues investigated in this survey, and listed above. As such, responders were classified as having minor (<25), low intermediate (25– <50), high intermediate (50– <75) and major (75–100) concerns for any aspect investigated. To better understand the decrease in the number of surgeries, the difference in the number of surgeries between the two investigated periods (before/during COVID-19) was calculated as percentage of the baseline pre-COVID-19 practice.

Univariate analysis was performed to identify possible significant differences between countries and hospital types, when applicable. The Fisher exact test and the chi-square test were used to analyze categorical variables, and the Student *t* test to analyze continuous variables. A power analysis was not required, due to the descriptive character of the survey without pursuing a specific hypothesis.

### Ethical Approval and Data Protection

Consent was not separately required. Participant could cancel the survey session without data being stored at any part of the questionnaire. The survey included a preliminary introduction regarding the nature of the study and an opt-out option asking to formally agree with the participation in this survey. Data protection/privacy policy was clearly provided by the survey platform, it applies to all data recorded using this survey (https://www.surveymonkey.com/mp/legal/privacy-policy/) and were viewable at any time by the participant. The study adhered to the 2016 version of the General Data Protection Regulation (GDPR) applicable in Europe since 2018 (https://gdpr-info.eu/). Data with potential personal data protection risk (such as the last five digits of the work/office phone number) were planned to be deleted from all files and platforms, if a follow up survey was not to be realized. In case of follow up data being acquired and analyzed; the corresponding numbers will be deleted after final data analysis.

## Results

### Participating Neurosurgeons

Two hundred and four questionnaires were completely filled out and their answers were included in this analysis. Responders from Europe, North and Central America, Asia, and Africa contributed to the global outreach of this research project, which was aimed to go beyond the borders of European countries as per the intention of its sponsor, the EANS. Excluding incomplete surveys, 204/205 were included for analysis resulting in a completion rate of 99.5% (versus the estimated completion rate of 52% initially calculated by the software). A response rate could not be accurately calculated based on the multimodality distribution utilized for this survey which was also open to non-members of the EANS; however, the most appropriate figure was estimated to be ranging between 5 and 10%, hence slightly higher than the response rate of previous surveys distributed worldwide among neurosurgeons (204 responses out of 1,800 members for the EANS survey vs. 1,294 responses out of 49,000 members for the World Federation of Neurosurgical Societies survey) (20–22). Table 1 provides detailed raw data from our survey.

**Table 1:** Individual perception of the crisis and crisis management.

**Numeric Scale Slider; No. (%)**	** <25% Minor**	**25– <50% Low-Intermediate**	**50– <75% High-Intermediate**	**75–100% Major**
Concerns regarding personal health	89 (44.3)	27 (13.4)	54 (26.9)	31 (15.4)
Concerns regarding families' health	47 (23.3)	34 (16.8)	45 (22.3)	76 (37.6)
Afraid of losing your job	154 (77.8)	15 (7.6)	21 (10.6)	8 (4)
Economic threat to practice	96 (48.2)	35 (17.6)	30 (15.1)	38 (19.1)
Private economic threat	77 (38.3)	48 (23.9)	43 (21.4)	33 (16.4)
Assumed financial loss	32 (29.4)	39 (35.8)	25 (22.9)	13 (11.9)
Increase in telemedicine	79 (39.3)	33 (16.4)	32 (15.9)	57 (28.4)
Concern that patients on the waiting list might deteriorate	47 (23.3)	47 (23.3)	65 (32.2)	43 (21.2)
**Yes/No Questions; No. (%)**		**Yes**		**No**
Institution adequately prepared		134 (65.7)		70 (34.3)
Feel adequately protected		101 (49.5)		103 (50.5)
Do you assume financial loss		122 (60.4)		80 (39.6)
Change internal communication		151 (74.4)		52 (25.6)
Ready to assume “foreign” duties		134 (66)		69 (34)
Already providing “foreign” duties		38 (18.7)		165 (81.3)
Feel adequately capable to do so		90 (44.3)		113 (55.7)
Neurosurgery staff rotated to “foreign” duties		91 (45)		111 (55)
Continue providing elective services				
Outpatient		29 (14.5)		171 (85.5)
Surgery		24 (12.4)		169 (87.6)
Sufficient preparation by Government and MOH		96 (47.5)		106 (52.5)
Sufficient preparation by local authorities		95 (46.8)		108 (53.2)
Sufficient preparation by hospital management		108 (53.2)		95 (46.8)

*MOH, Ministry of Health; No., Number of patients; %, Percent. Description of each question was modified to fit in a proper table format. The total amount of participants might differ between questions, given variation in answer rates for each question. Estimation of assumed financial loss was only provided by participants assuming some kind of financial loss*.

**Figure 1:**
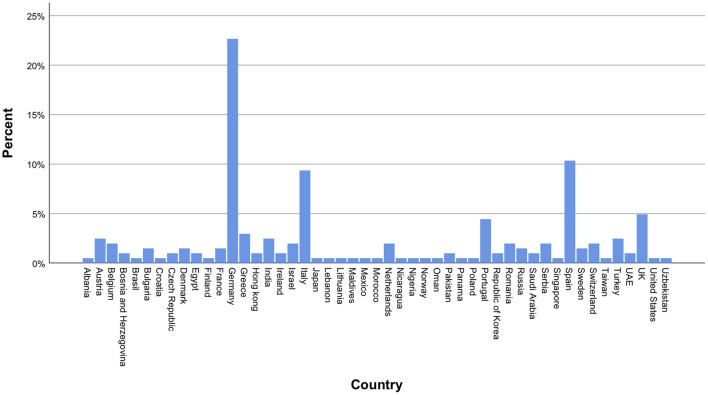
International distribution of participating neurosurgeons. The majority of participants were from Germany, Spain, Italy and the United Kingdom.

**Figure 2:**
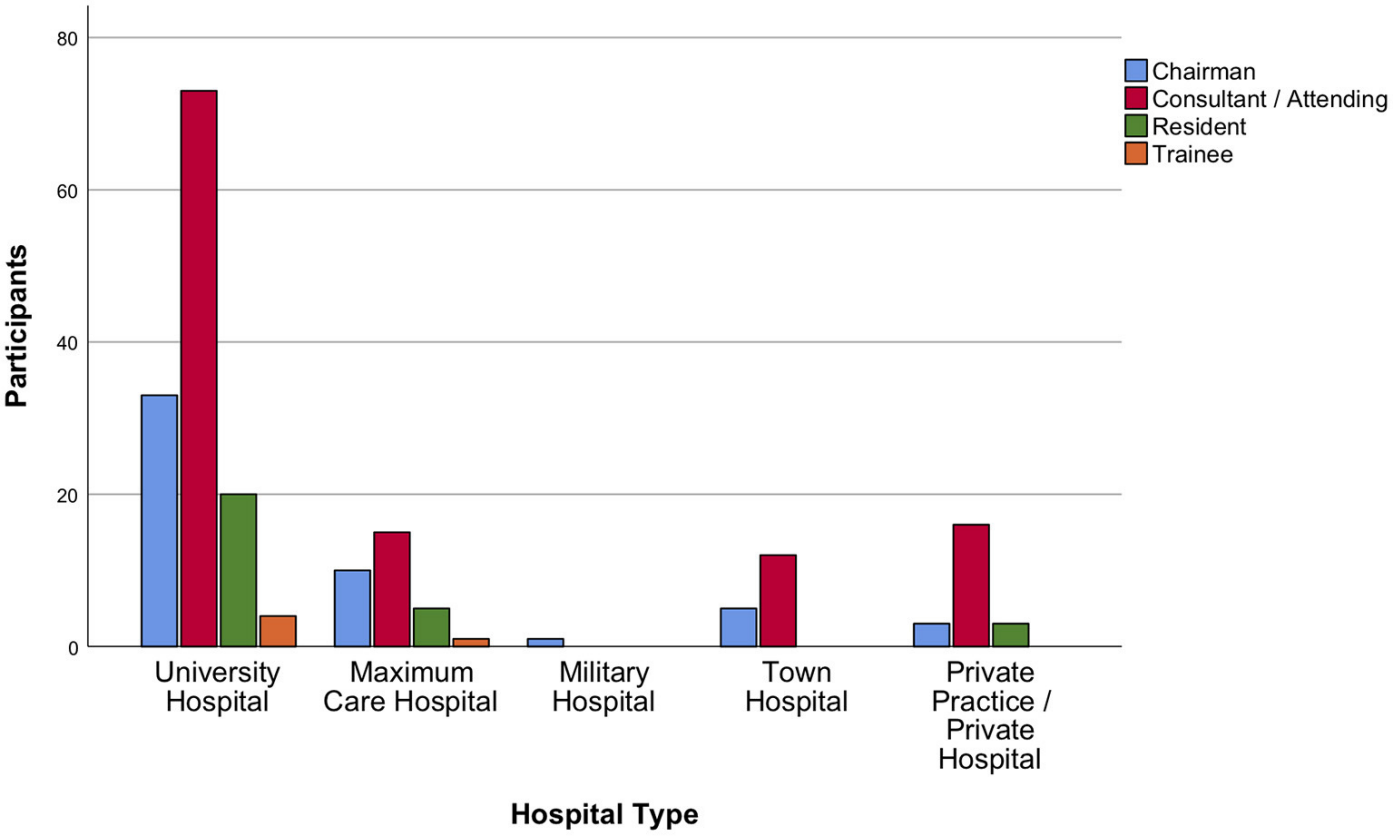
Distribution of participants among hospital types. Most contributions were received from university hospital neurosurgeons. Consultant/attending neurosurgeons and department chairmen took the survey more frequently than trainees and residents.

Most contributions originated from Germany, followed by Spain, Italy, and the United Kingdom (UK), thus providing a balance between nations mildly affected (Germany and the UK) vs. those severely affected (Spain and Italy) by the first wave of the pandemic. Neurosurgeons from Lombardy accounted for 5% of total responses. A detailed list of countries is displayed in Figure 1. Overall, 64.5% of participants were neurosurgeons working in a university hospital (Figure 2). The survey was predominantly completed by consultant/attending neurosurgeons and department chairmen, which accounted for 57.9 and 25.7% of participations, respectively (Figure 2). Overall, EANS members answered 73% of the surveys.

### Neurosurgical Shutdown in Figures

**Figure 3:**
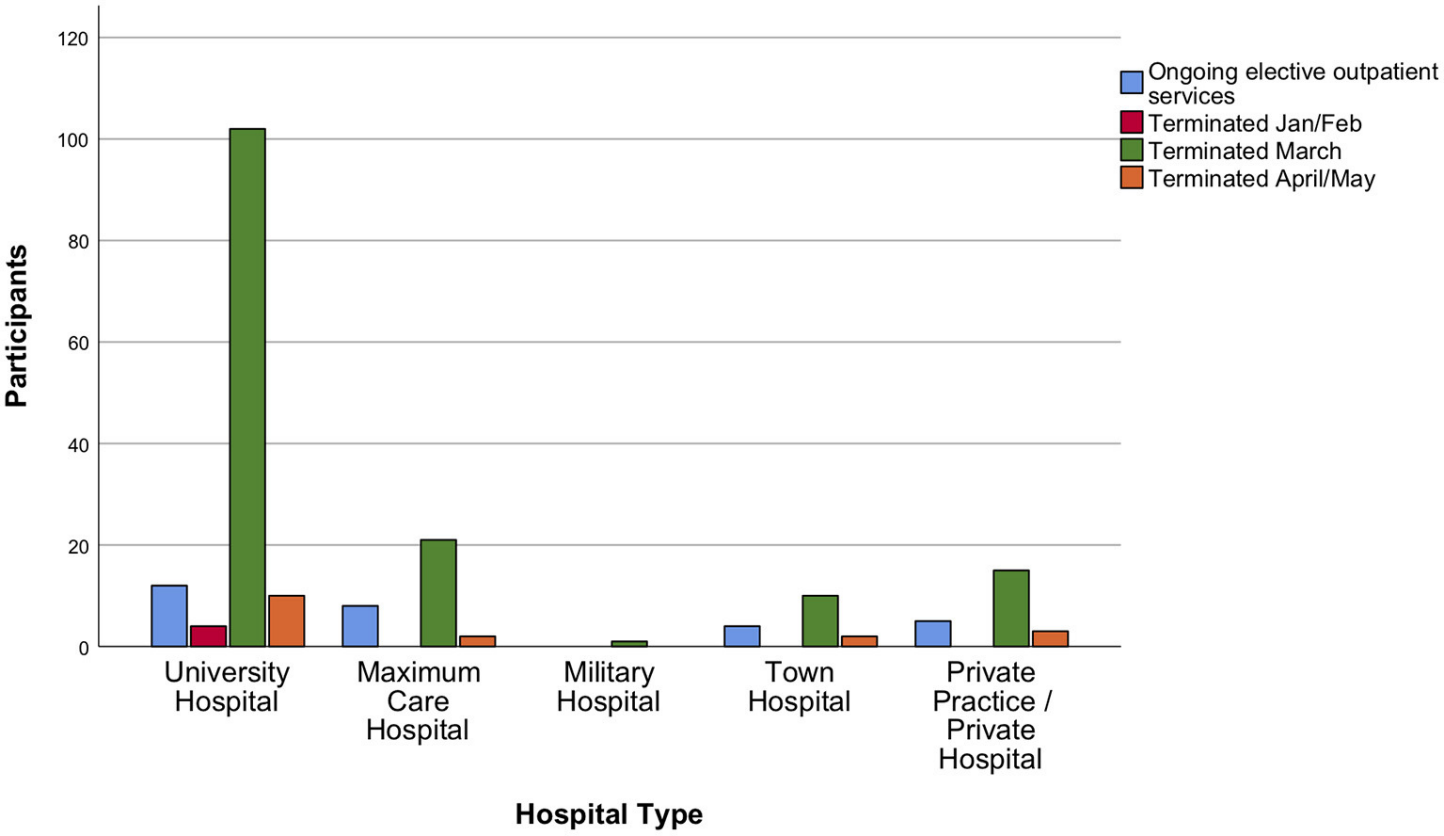
Outpatient elective services during the acute phase of the crisis. The majority of participants confirmed termination or reduction of elective outpatient services to a minimum in their institution at some timepoint in March 2020.

**Figure 4:**
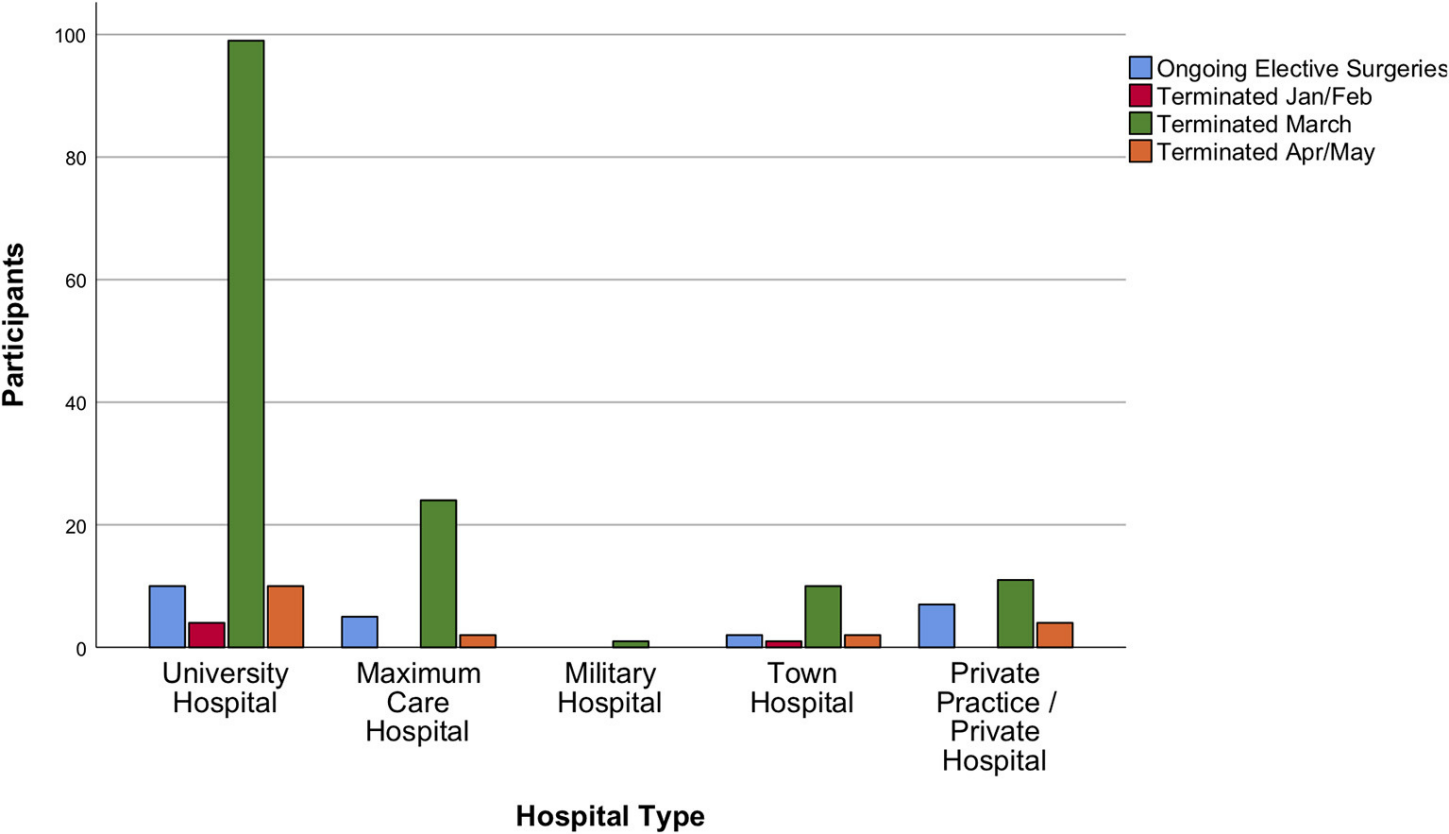
Surgical elective services during the acute phase of the crisis. Elective surgical services were also confirmed to be terminated or reduced to a minimum at some timepoint in March 2020 by participating neurosurgeons.

The survey demonstrated that 85.5% and 87.6% of neurosurgical departments had either terminated or reduced to a minimum all types of elective service (Figures 3, 4). Most neurosurgeons reported that shutdowns of their elective practice had started even before lockdowns were ordered on their national territory (75% of responders indicated shutdowns were started in early March 2020). Those measures had been taken in agreement to hospital policy, and only in 20% of the cases were based on a precautionary decision taken solely at departmental level. The responses collected revealed that personnel shortages, either in terms of available nurses, anesthetists, or surgeons, had relevant impact on the earlier shutdown of neurosurgical elective activities.

Only 2.5% of responders stated that patients did not understand nor seemed to accept these measures. More than half of responders (53.5%) expressed major or high intermediate concerns that patients on the waiting list might significantly deteriorate while waiting for the shutdown measures to be eased (Table 1).

**Figure 5:**
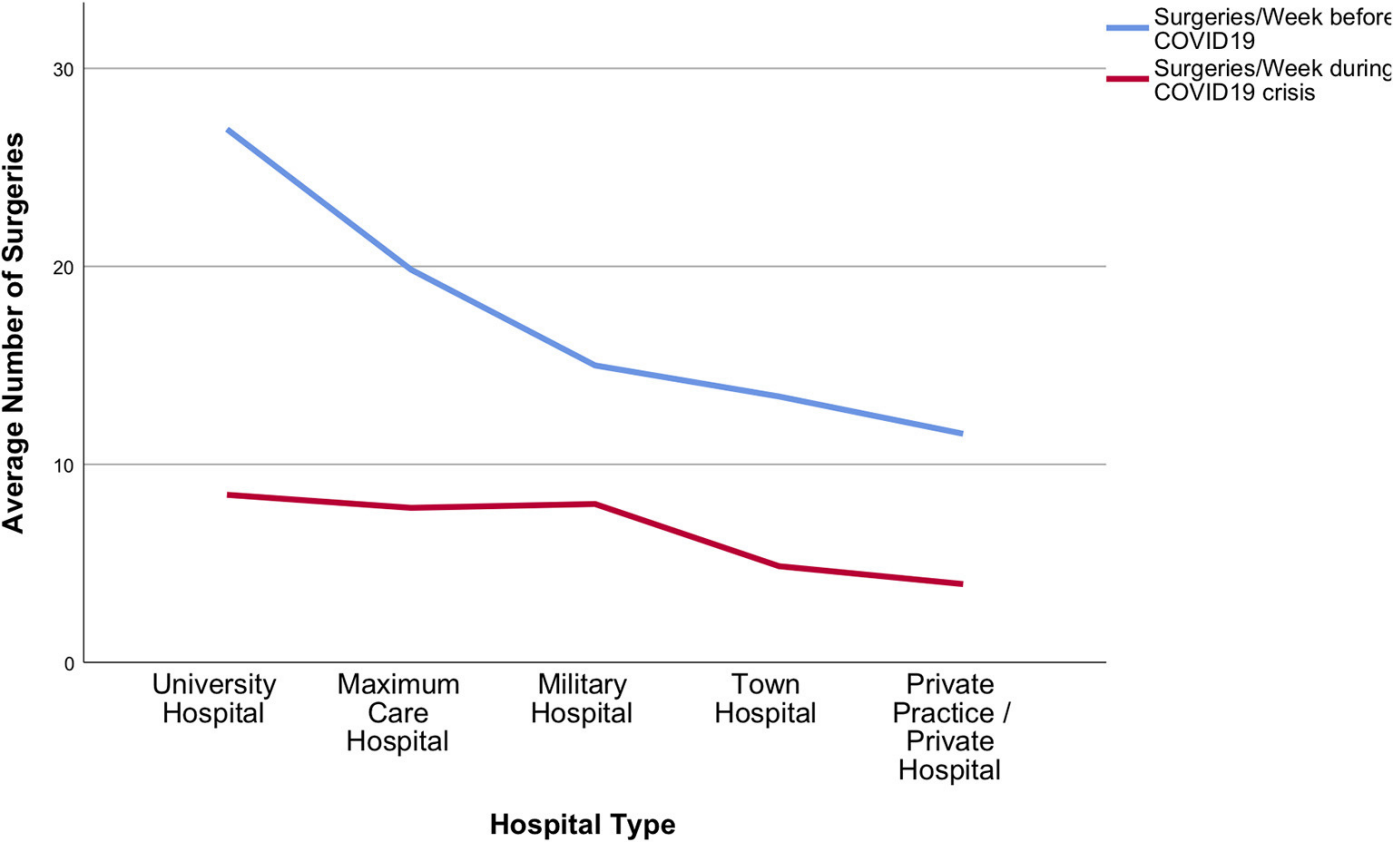
Surgical services during the acute phase of the crisis. Total average number of surgeries dramatically decreased during the COVID-19 pandemic in all participating hospital types.

**Figure 6:**
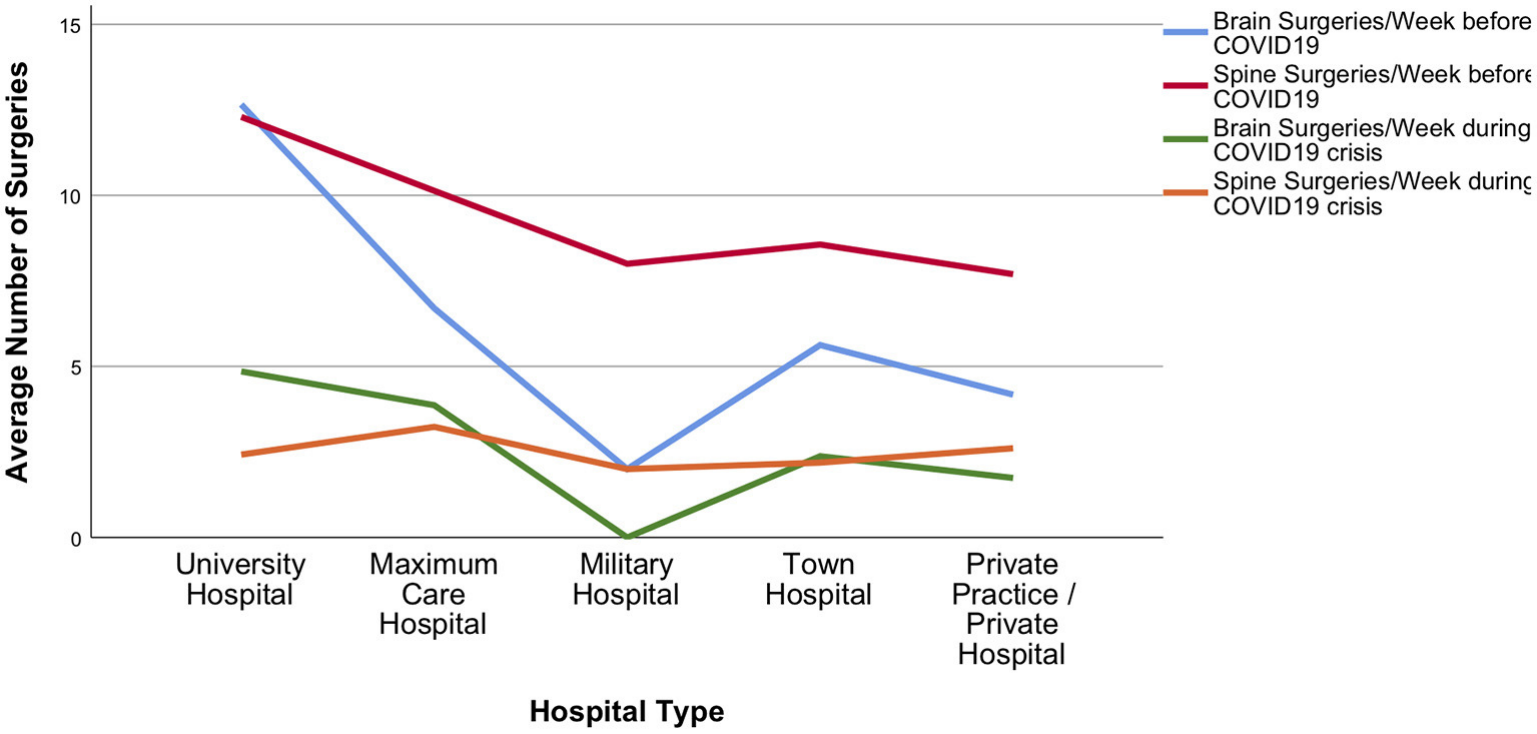
Brain and spine surgery during the acute phase of the crisis. Detailed presentation of decline in average brain and spine surgeries among different hospital types during the COVID-19 pandemic.

In relation to the pre-COVID-19 practice, the total volume of neurosurgical activities was on average reduced by 68 ± 21.8%. Cranial surgery decreased by 54 ± 31.3%, spine surgery by 71 ± 31%, and emergency surgery by 35 ± 31.9%. Decline in average weekly surgery numbers is shown in Figures 5, 6.

### Changes in Communication Styles and Methods

The survey showed that 74.4% of neurosurgeons had changed their internal communication routine with other local and international colleagues: this included job-planned clinical activities, such as daily ward rounds and weekly multidisciplinary meetings, but also scheduled academic or professional activities, such as conferences. Most free text elaborations revolved around the importance of electronic communication (either via emails or other forms of instant messaging and videoconferences) to ensure sufficient social distance and avoid complete cancellation of those vital components of our regular clinical and non-clinical activities. Telemedicine was reported to have increased more than 50% in 44.3% of neurosurgical departments included in this survey (Table 1). While responders from Lombardy provided experiences from the Italian epicenter of the pandemic, neurosurgical practice in the rest of the country had been much spared by its first wave, as such they reported significantly less change in daily routine and communications (*p* = 0.02).

### Perception of Health, Safety, Economic Risks and Crisis Management

When asked to rate their concerns regarding personal health during the pandemic, 44.3% of participants declared minor concerns, compared to 37.6% who reported major concerns regarding their families' and relatives' health (Table 1).

A total of 65.7% of the responders stated that their institution was adequately prepared for this crisis, however only 49.5% felt adequately protected by their institution. Answers were discordant about perception of measures and regulations undertaken by politicians and regional authorities: less than half of the neurosurgeons felt sufficiently prepared by their local authorities (46.8%), and their government and ministry of health (47.5%).

Some responders mentioned lack of advice and training (53%), others limited patient testing for COVID-19 (56%), and the majority (85%) complained of shortages in personal protection equipment (PPE) as reason for this answer. Participants reported a total of 187 neurosurgical staff members having been infected with SARS-CoV-2 up until May 2020.

Co-workers' reaction to the crisis was mainly perceived to be professionally appropriate by 44.8% of responders; only a minority reported an unjustified tendency towards staying home (8.4%).

### Perception of Financial Security

Professional career safety (e.g., losing the job as a neurosurgeon) appears to only play a marginal role, in fact 50% of participants had no concerns at all, while 0.5% with highest concerns and the largest group of responders (85.4%) described minor or low intermediate distress regarding this matter. However, financial concerns regarding private and hospital-based neurosurgical practice were clearly stated, with 4 and 5.5% stating highest concerns, and only 16.4 and 20.5% having no concerns at all, respectively. Of note, 37.8 and 34.2% reported high intermediate and major worries, respectively. Furthermore, 60.4% did anticipate experiencing some kind of financial loss due to COVID-19, 34.8% estimated their financial loss to be more than 50%, and 0.9% anticipated to be left without any sort of financial revenue during the pandemic (Table 1).

### Unequal Impact of COVID-19 on Private and Academic Workforce

A sub-analysis meant to identify differences among responders with regards to financial security revealed an unequal impact of the pandemic on neurosurgeons conducting private practice only, vs. those with any other type of working appointment. Specifically, private practice and town hospital employees were more concerned about losing their job, compared to university hospital (*p* < 0.001). Compared to any other group of responders, academic neurosurgeons also seemed to be more prone to adapt to changes in internal communication (*p* = 0.09) and to accept better the use of telemedicine (*p* = 0.031). Notably, university hospital neurosurgeons described less ongoing elective outpatient (*p* = 0.005), and surgical services (*p* = 0.015), nonetheless they also reported higher chances of rotating to ED/COVID-19 services than anyone else (*p* < 0.001). Differences among hospital types in declining surgery numbers are demonstrated in Figures 5, 6. No significant differences or trends were detected when comparing responders on their level of seniority because most participants belonged to the same group: consultants/attendings.

### Self-Confidence When Attending COVID-19 Duties

Most neurosurgeons (66%) seemed ready to fulfill non-specialty-related COVID-19 duties in ICU or ED: 18.7% of survey participants were already providing such services, whereas 45% stated that neurosurgical personnel were expected to rotate soon to such duties. However, only 45% felt adequately capable to do so based on previous training completed during their residency (Table 1).

## Discussion

Ever since the beginning of COVID-19 outbreak there was no doubt that this unprecedented crisis would have had a substantial impact on all healthcare practitioners. Surgical specialties, such as neurosurgery, were immediately affected by direct and indirect consequences of this pandemic (23). In fact, its dimensions forced neurosurgeons to quickly rethink and timely reorganize their practice. This survey's objective was to shed some light on how many and how severe the implications of COVID-19 had been for European and international neurosurgeons reached by the EANS. Within a short time of about 6 weeks, over 200 neurosurgeons participated in this qualitative study, which recorded a significant contribution from countries severely affected by the initial wave of the pandemic. Each survey question was designed to learn insightful lessons from their frontline experience, which will be useful to better prepare for the future evolution of this healthcare crisis. Completion rate was rather high with 204/205 (>99%) completed surveys. Response rate could not be exactly calculated based on the used means of distribution. An estimation at 5–10% was comparable, even higher, than formerly published neurosurgical surveys (20–22).

### Organizational Perspective

Despite being served by modern hospital networks, counting on a well-organized welfare, European countries were severely affected by the pandemic, and answers from European participants barely differed from those of responders from low- and middle-income countries. Not only this testifies how powerful the COVID-19 outbreak has been so far, effectively leveling all healthcare systems, but also it highlights that the countermeasures adopted were very similar everywhere, being translated from well-established working frameworks of medical contingency planning and catastrophic disaster response.

One of the most relevant points is that the early termination of any elective neurosurgical service by about 70% was very diffused regardless of the nationality and type of hospital practice of the responders. Not surprisingly, the concerns raised by a large proportion of responders regarding the risks of triaging patients and rationing the neurosurgical offer are in keeping with those recently reported by others (2, 9, 12, 18, 24–28). Furthermore, it should be noted that the extended lockdown period, and the justified patients' anxiety of getting infected with SARS-CoV-2 when attending hospitals, might have delayed access to care for most pathological conditions. This finding had already been described for acute cardio- and cerebrovascular emergencies, hence the neurosurgical community must reflect on the dramatic consequences in terms of morbidity and mortality of timely managing otherwise treatable diseases, which could potentially escalate to more advanced, incurable stages (29). Beside the clinical challenges of treating more advanced conditions, the dimensions of their economic and financial sequelae to public healthcare systems also remain unclear. Although this last aspect is outside the scope of the present survey, it represents one of the research questions eventually generated by the analysis of data collected.

The fast and multilayered changes implemented to face the pandemic have also fostered the adoption of a variety of electronic and online means of communication (30, 31). Such trend has been well captured by our survey, which however did not investigate how such organizational change affected the effectiveness and quality of care. Some of the aspects not allowing for those types of inferences relate to the heterogeneous national and international regulations, medico-legal implications, and financial compensation among the countries included in this study.

### Trends Across Countries

National and regional breakdown to identify clusters of responders were made possible by secondary analysis of raw data: for instance, neurosurgeons from Lombardy accounted for a high response rate indicating that this survey had great appeal on those colleagues most affected by the pandemic. Of note, this survey demonstrated a number of profile- and age-related trends. For instance, while senior German neurosurgeons tended to feel adequately prepared to assume duties as ED or ICU doctors based on their training during residency (*p* < 0.001), this was not the case for more junior German surgeons who had been brought up without hands on experience during their ICU rotation.

The trends identified indicate that neurosurgeons come from very different backgrounds and clinical practice: some of those trends are likely to be related to the impact of the pandemic (e.g., high or low mortality rate attributable to COVID19), but most of them are due to the international variation in terms of neurosurgical training, and the remuneration of fully trained neurosurgeons. All those aspects might strongly differ between countries, and even more so across continents. Although interesting, the trends emerging from the analysis of data collected should be reviewed with caution because this survey adopted a self-selection enrollment, which resulted in different groups size per nations.

### Individual Perception of the Crisis

Of note, the results of this survey suggested that neurosurgeons care more about their families' health than their own. As healthcare professionals, neurosurgeons have handled the crisis in a professional manner, despite the special risk profile associated with the exposure to infective aerosols (such as those produced during high-speed drilling, or generated bipolar/monopolar cautery, not to mention while exposing the rhinopharyngeal mucosae during trans-sphenoidal surgery) (32, 33). As many other professionals, neurosurgeons did assume substantial financial and organizational implications for their hospital and private practices. This might be directly linked to various factors, including the lack of working stability for those solely employed in private practice, the increased difficulties in accessing research funding (for instance due to research grants being diverted to other areas of COVID-19 research) for pure academics and surgeon-scientists, the need for redeployment of personnel and resources for those working in public hospitals.

Furthermore, one relevant result of this survey is that it demonstrated how the neurosurgical community is not equally affected by the financial threats caused by the pandemic. Only 33% of responders expected to have a significant financial loss, the survey demonstrated that this risk is particularly high for neurosurgeons working only in private practice. Those are in fact exposed more than others to financial turmoil and are effectively left without much protection if prevented from making a living out of elective surgery.

### Potential for Improvement

Data from this survey indicate the importance of international cooperation, rapid preparation and circulation of robust recommendations, and support for frontline professionals, especially from severely affected countries. To this extent the EANS efforts to inform, connect and update members and non-members from all over the world are worth mentioning. In addition to the EANS president's statement (34), the EANS IMC (35) and the EANS Ethico-Legal Committee (2) produced relevant documents to help addressing critical aspects of our practice, namely the need for PPE and psychological support, or the ethical dilemmas caused by triaging and rationing access to care. The present survey complements well the previous contributions. In fact, it allowed a step forward in the understanding of the snapshot provided by Mathiesen et al. (2), which had a departmental perspective, by researching COVID-19 impact on the personal sphere of individual neurosurgeons.

The COVID-19 pandemic affected neurosurgery as a specialty, and neurosurgeons as independent healthcare practitioners. As any other crisis, this outbreak brought up unprecedented challenges, and exposed healthcare practitioners to new risks; however, it also highlighted new opportunities to strengthen the neurosurgical community and improve daily practice. Technologies that were already available but not fully employed in daily practice, such as telemedicine, were rediscovered, whereas other areas of digital medicine were fully exploited to expedite virus detection and clinical diagnosis. Neurosurgeons became more flexible with their working schedule, adopted new communication styles, and adapted to the use of PPE in clinical wards and operating rooms.

### Limitations

The results of this survey are limited by the number of participants, their origin, and intentionally by the subjective statements of single individuals. Although this study fulfilled its goal of being representative of EANS-affiliated neurosurgeons, one should be careful of generalizing those results to the entire international community of neurosurgeons. While the majority of questions were designed as multiple-choice, free text could be submitted in various subsections of the survey hence the total amount of information provided might be hardly comparable. Although the study design focused on the potential for the data collected to be representative of both the professional and individual aspects of the COVID-19 pandemic, the large number of responders would warrant scrutiny toward risk of biases. Since more than two hundred European and international neurosurgeons participated, this survey is subject to individual variations among participants, their personal circumstances, stressful working pattern and individual aversion to uncertainty might have heavily influenced their answers. Detailed demographics such as the seniority of responders according to the number of years of neurosurgical practice were not specifically inquired but could be extrapolated by the stratification based on the participants' working position (such as Chair, Consultant or Trainee). However, one should also consider that in pragmatic qualitative studies with an inductive approach (meant to explore the characteristics of the problem, not that of the subjects), representativeness is not the priority (36). This was precisely the intention of this study: not to question official numbers or produce new ones, but simply to document the perceptions of neurosurgeons as professionals and individuals in order to help EANS assisting them better in the forthcoming of this healthcare crisis. Follow up surveys on mid- and long-term after-effects of the crisis have already been projected.

## Conclusion

Not only this survey demonstrated that neurosurgeons have a diffuse fear of exposing patients and families to contagion with SARS-CoV-2; but it also highlighted that some of the responders were more susceptible to unexpected turmoil, both in terms of psychological burden regarding individual job security, and financial instability related to lack of continuity in employment-generated income. Therefore, to navigate through this crisis and the ones ahead of us, neurosurgeons should leverage on the lessons learnt, and define innovative strategies to cope with and limit the abovementioned risks.

## Data Availability Statement

The original contributions presented in the study are included in the article/Supplementary Material, further inquiries can be directed to the corresponding author/s.

## Ethics Statement

Ethical review and approval was not required for this study in accordance with the local legislation and institutional requirements.

## Author Contributions

SR and HC initiated the project and drafted the survey. SR, HC, MG, CZ, MB, and AG contributed to finalizing the survey. CZ, MB, and AG assisted with tables and figures. SR performed the statistical analysis. MB and HC supervised manuscript production. MG and SR finalized the manuscript. All authors contributed to the article and approved the submitted version.

## Conflict of Interest

The authors declare that the research was conducted in the absence of any commercial or financial relationships that could be construed as a potential conflict of interest.

## Publisher's Note

All claims expressed in this article are solely those of the authors and do not necessarily represent those of their affiliated organizations, or those of the publisher, the editors and the reviewers. Any product that may be evaluated in this article, or claim that may be made by its manufacturer, is not guaranteed or endorsed by the publisher.
