# Supplementary material for: Unequal Impact of COVID-19 on Private and Academic Neurosurgical Workforce: Results of an International Survey
Source: Front Surg. 2021 Oct 1;8:749399. doi: 10.3389/fsurg.2021.749399 (PMC8517237; doi:10.3389/fsurg.2021.749399)
Supplement: Supplementary file 1 [file Data_Sheet_1.PDF]

# Neurosurgeons in Times of Crisis – Questionnaire regarding Neurosurgery across Europe during the COVID-19 Crisis

Dear colleagues and friends,

in times of crisis such as the one we are all experiencing now with COVID-19, most of us are already affected in their daily professional life. This situation is novel to all of us. The health system has to deal with the crisis, but every one of us is also affected personally. We hope that we can learn how to deal with such a crisis and use this experience to improve things for the future. The questionnaire is anonymized and consists of personal as well as few department-related questions. Please kindly complete all the questions to help us analyze the impact of this crisis on us and our practice.

Follow-up questionnaires in about 3-, 6- and 12-months will help us estimate the mid and long-term impact. International EANS members and colleagues are also welcome to participate. Please share with your colleagues and non-member Neurosurgeons.

Thanks to you, stay healthy, good luck

1. Country

2. City

3. No. of Neurosurgery Departments in your City

4. Hospital Type

- ◯ University Hospital
- ◯ Town Hospital
- ◯ Maximum Care Hospital
- ◯ Private Practice / Private Hospital
- ◯ Military Hospital

## 5. Size of Neurosurgical Department

No. of Beds

No. of Consultants

No. of Residents/Trainees

No. of Surgeries per Year

## 6. Your Occupation

- Chairman
- Consultant / Attending
- Resident
- Trainee
- Medical Student

## 7. Are you an EANS Member?

- YES
- NO

IF NO: Other Memberships:

## 8. Please enter the LAST FIVE DIGITS of your office/work phone number

*[To help anonymously and distinctly match your answers with follow up surveys, if you kindly participate in the future. Otherwise the number will be deleted after final data evaluation]*

## 9. Do you have concerns regarding your personal health?

NONE — MAJOR

10. Do you have concerns regarding the health of your family and relatives?

NONE MAJOR

11. Is your institution adequately prepared for such a crisis?

- YES
- NO

IF NO, WHY?

12. Do you feel adequately protected by your institution in such a crisis?

- YES
- NO - IF NO: WHY NOT, what is lacking:
- Advice and Training
- Patient testing for COVID
- Safety Equipment (Masks, Suits, etc.)

13. How afraid are you of losing your job as a Neurosurgeon?

NO FEAR MAJOR FEAR

14. How concerned are you about an economic threat to your neurosurgical practice?

NOT AT ALL MAJOR

15. How concerned are you about an economic threat to your private economic stability?

NO AT ALL MAJOR

16. Do you assume to experience personal financial disadvantages due to the crisis?

- YES
- NO

IF YES, financial loss to what percentage (0-100%)

17. To what extent did you increase telemedicine (tele-consultation or direct video-sessions with patients) ?

NOT AT ALL — INTENSLY

18. Did you change your internal communication with colleagues for ward rounds, conferences etc.?

- YES
- NO

IF YES: Which tools did you use?

19. Do you feel ready to assume divergent functions as an emergency doctor in case required?

- YES
- NO

IF NO, WHY?

20. Are you already personally providing emergency medical service instead of neurosurgical practice?

- YES
- NO

IF YES, HOW OFTEN/FOR HOW LONG

21. Do you feel adequately capable of providing emergency and ICU services based on specific training during your residency?

- YES
- NO

22. Neurosurgery personnel rotated to emergency services / COVID-19 treatment

- YES
- NO

23. Date regular outpatient service was terminated in your department

[If you are still providing regular elective services use 01.01.2001]

Date

MM/DD/YYYY

24. Do you feel patients understood the need to terminate elective service?

- YES TOTALLY
- YES BUT NOT ALL
- YES ONLY A FEW
- NO

25. Date Regular Elective Surgeries were terminated / only Emergencies in your department

[If you are still providing regular elective services use 01.01.2001]

Date

MM/DD/YYYY

26. Average No. of Surgeries/Week in your department before COVID19

27. Average No. of Brain Surgeries/Week in your department before COVID19

28. Average No. of Spine Surgeries/Week in your department before COVID19

29. Average No. of Emergency Surgeries/Week in your department before COVID19

30. Average No. of Surgeries/Week NOW (04/2020) in your department during COVID19 crisis

31. Average No. of Brain Surgeries/Week NOW (04/2020) in your department during COVID19 crisis

32. Average No. of Spine Surgeries/Week NOW (04/2020) in your department during COVID19 crisis

33. Average No. of Emergency Surgeries/Week NOW (04/2020) in your department during COVID19 crisis

34. If regular surgeries were cancelled before political obligatory shut down, for what reason:

- Hospital policy
- Personnel shortage nurses
- Department precautionary measures
- Personnel shortage surgeons
- Personnel shortage anesthetists

35. How concerned are you that your patients on the waiting list might relevantly deteriorate?

NOT AT ALL — MAJOR

36. No. of corona infected neurosurgical personnel in your department

0 — 100

37. Do you feel your government and ministry of health sufficiently prepared you for this crisis?

- YES
- NO

38. Do you feel your local authorities sufficiently prepared you for this crisis?

- YES
- NO

39. Do you feel your hospital management sufficiently prepared you for this crisis?

- YES
- NO

40. Reaction of your co-workers and personnel to the crisis in your opinion:

- Professional with major concerns
- Professional with little concern
- Major concerns with tendency to stay home
- All of the above

41. In your opinion; what elective/semi-elective neurosurgical patients cannot be postponed despite the crisis

42. Improvement suggestions regarding crisis management concerning Neurosurgery
